# Unusual cause of weight loss in a patient with HIV–hepatitis C virus coinfection

**DOI:** 10.4103/2589-0557.75004

**Published:** 2010

**Authors:** A. Gogia, A. Kakar

**Affiliations:** Department of Medicine, Sir Ganga Ram Hospital, Rajinder Nagar, New Delhi - 110 060, India

**Keywords:** HIV, interferon α, thyroiditis, weight loss

## Abstract

Recombinant interferon α (IFN α), alone or in combination, is used extensively in the treatment of hepatitis C infection. IFN therapy is not free of side-effects and autoimmune thyroiditis is one of its rare side-effects. We present here a case of a patient with hepatitis C virus–human immunodeficiency virus coinfection on interferon therapy who presented with significant weight loss. He was found to have IFN-related autoimmune thyrotoxicosis and responded to antithyroid drugs and propanolol. Therefore, this case highlights that IFN-induced thyroiditis is an unusual side-effect and that during treatment, a thyroid-stimulating hormone assay should be performed at regular intervals (every 8–12 weeks).

## INTRODUCTION

Recombinant interferon α (IFN α), alone or in combination, is used extensively in the treatment of hepatitis C infection. IFN therapy is not free of side-effects and autoimmune thyroiditis is one of its rare side-effects. IFN has a direct inhibitory effect on thyroid hormone synthesis, metabolism and release. A genetic predisposition to thyroid autoimmune disease is probably necessary for the development of thyroid disease in patients on IFN.[1,2] The prevalence of thyroid disease during IFN therapy is highly variable, ranging between 1 and 35% in different studies.[3]

## CASE HISTORY

A 31-year-old male was diagnosed as a case of pulmonary tuberculosis with acquired immunodeficiency syndrome in March 2003. He was started on 4-drug antitubercular treatment. At that time, his weight was 61 kg, CD4 count was 62 cells/ml and human immunodeficiency virus (HIV) RNA viral load was 512,000 copies/ml. During therapy for tuberculosis, he developed hepatitis within 2–3 weeks. On evaluation, he was found to be positive for hepatitis C virus (HCV) (Genotype 1), with an HCV RNA viral load of 205,000 copies/ml. Highly active antiretroviral therapy (HAART) (Zidovudine, Lamivudine and Efavirenz) was started after 3 months of Anti-tubercular treatmentIFN α and ribavirin (1000 mg/day) (for 48 weeks) were also started with HAART as part of treatment for HCV coinfection. Before starting IFN, his thyroid profile was normal and thyroid antibodies were negative. The patient improved clinically and the viral load decreased. He gained weight in 2004 and his weight became 74 kg, with undetectable HIV RNA viral load, CD4 count of 375 cells/ml and HCV viral load of <500 copies/ml. In July 2005, the HCV viral load increased to 930 copies/ml, HIV RNA load was <400 copies/ml and CD4 count was 969 cells/ml. He had continued HAART and was put on pegylated IFN α along with ribavirin for HCV relapse. He remained stable on this treatment. However, in December 2005, he complained of weight loss of 15 kg in about 6 months. He was evaluated for his weight loss. His thyroid profile showed TSH <0.01 Miu/L, FT3 30.8 mmol/l, FT4 4.6 pmol/l and antithyroperoxidase (TPO) positive. The radioactive uptake scan revealed increased uptake. Because only three doses of IFN were left, it was decided to continue the IFN for HCV treatment. Thus, a final diagnosis of IFN a-induced thyroiditis leading to weight loss with HCV–HIV coinfection was made. He was initially given propanolol but his symptoms were not controlled and thus, later, propylthiouracil was added. The patient gained weight over next few weeks, which went up to 70 kg (gain of 11 kg). The thyroid profile normalized on a maintenance dose of propylthiouracil 30 mg/day.

## DISCUSSION

Although the prevalence of thyroid dysfunction in patients treated with IFN α is 1–35%, the prevalence of thyrotoxicosis in HCV patients treated with IFN α has been reported to occur in 2–3% of treated patients only.[3] Women are more susceptible than men to develop IFN related-thyroid dysfunction, having a relative risk that is 3- to 7-fold higher, as reported in most studies.[1,3] It has been suggested that HCV might share partial sequences in a few amino acid segments with thyroid tissue antigens.[1] The long-acting pegylated IFN α molecule has similar rates of thyroid side-effects as with IFN α. The development of thyroid disease does not seem to be related to the dose of IFN α. In contrast, the duration of IFN α treatment has been related to the occurrence of thyroid dysfunction in some studies.[1] However, two or more cycles of IFN α therapy did not increase the risk of the development of thyroid disease. Ribavirin is the other drug used in combination with IFN α, as in our case. Patients treated with both drugs have a 4.3 relative risk to develop thyroid dysfunction, likely as a consequence of enhancement of the Th1 immune response that induces cell-mediated cytotoxicity.[4–6] A metaanalysis of the literature by Koh *et al*.[3] showed that about 50% of the patients with positive thyroperoxidase antibodies (TPO Abs) before IFN α treatment developed thyroid dysfunction in comparison with 5.4% in antibody-negative patients. The relative risk to develop thyroid dysfunction, mainly hypothyroidism, is 2- to 14-fold higher in patients with pre-existing positive TPO Abs compared with patients with negative antibodies.[1,7] Thyrotoxicosis is frequently mild and transient without overt clinical manifestations and may be diagnosed only by obtaining frequent thyroid function tests. The duration of destructive thyrotoxicosis is variable, ranging from a few weeks to a few months. Although isolated destructive thyrotoxicosis has occasionally been described, hypothyroidism often develops later. Some authors have reported complete recovery after withdrawal of IFN while others reported partial recovery. Before patients undergo treatment with IFN α therapy, it is suggested that serum TSH, FT4, Tg Ab and TPO Ab concentrations and perhaps a thyroid echography should be carried out to identify pre-existing thyroid dysfunction and autoimmunity.[1] During IFN α treatment, measurement of serum TSH concentrations should be carried out every 8–12 weeks. This is adequate to diagnose hypothyroidism but there is no sufficient literature to suggest the timing of thyroid function tests to detect thyrotoxicosis. When destructive thyrotoxicosis has been established, treatment with β-blocking agents is useful to control the symptoms and signs of thyrotoxicosis and discontinuation of IFN α is not required in most cases. IFN treatment may also induce Graves’ hyperthyroidism. The prevalence of this disorder is less frequent than other thyroid dysfunctions. The low prevalence of Graves’ disease during IFN α treatment may be related to the generalized suppressive effects of cytokines on the TH2 immune response. Suppressed serum TSH and elevated or normal FT4 and FT3 concentrations accompanied by elevated or normal thyroid radioiodine uptake value and positive thyroid antibodies confirm the diagnosis of Graves’ hyperthyroidism,[1] as was seen in our case. The approach to patients with Graves’ hyperthyroidism requires antithyroid drugs in mild cases, as was seen in our case, while in severe cases, radioactive iodine may be required. Remission of Graves’ disease is unlikely and requires long-term antithyroid drugs.[7]

## CONCLUSION

IFN-induced thyroid dysfunction is known but rare side-effect must be sought in patients with weight loss, with treatment involving β-blocking agents and/or antithyroid drugs. IFN therapy need not be discontinued in mild diseases.

## LEARNING POINTS

1. Interferon-induced thyroiditis is an unusual side-effect and Graves’ disease/thyrotoxicosis is even more unusual.
2. In chronic hepatitis C, systemic thyroid assessment should be performed before initiating IFN therapy, including clinical examination and measurement of TSH and anti-TPO Abs.
3. During treatment with IFN, a TSH assay should be performed at regular intervals (every 8–12 weeks).
4. IFN-induced Graves’ disease requires long-term treatment with antithyroid drugs in mild cases and radioiodine therapy in severe disease.
